# Effective Approach toward Selective Near-Infrared Dyes: Rational Design, Synthesis, and Characterization of Thieno[3,4-*b*]thiophene-Based Quinoidal Oligomers

**DOI:** 10.1021/acsami.2c18633

**Published:** 2022-12-12

**Authors:** Yuxuan Hei, Xinwei Zhang, Pengxing He, Eric Jiahan Zhao, Edison Tang, Valerii Sharapov, Xunshan Liu, Luping Yu

**Affiliations:** † Key Laboratory of Surface and Interface Science of Polymer Materials of Zhejiang Province, Department of Chemistry, Zhejiang Sci-Tech University, 928 Second Street, Hangzhou310018, China; ‡ Department of Chemistry and the James Franck Institute, The University of Chicago, 929 E57th Street, Chicago, Illinois60637, United States

**Keywords:** NIR dyes, quinoidal oligomers, thieno[3,4-*b*]thiophene, selectively controlled absorptions, transparent in the visible region

## Abstract

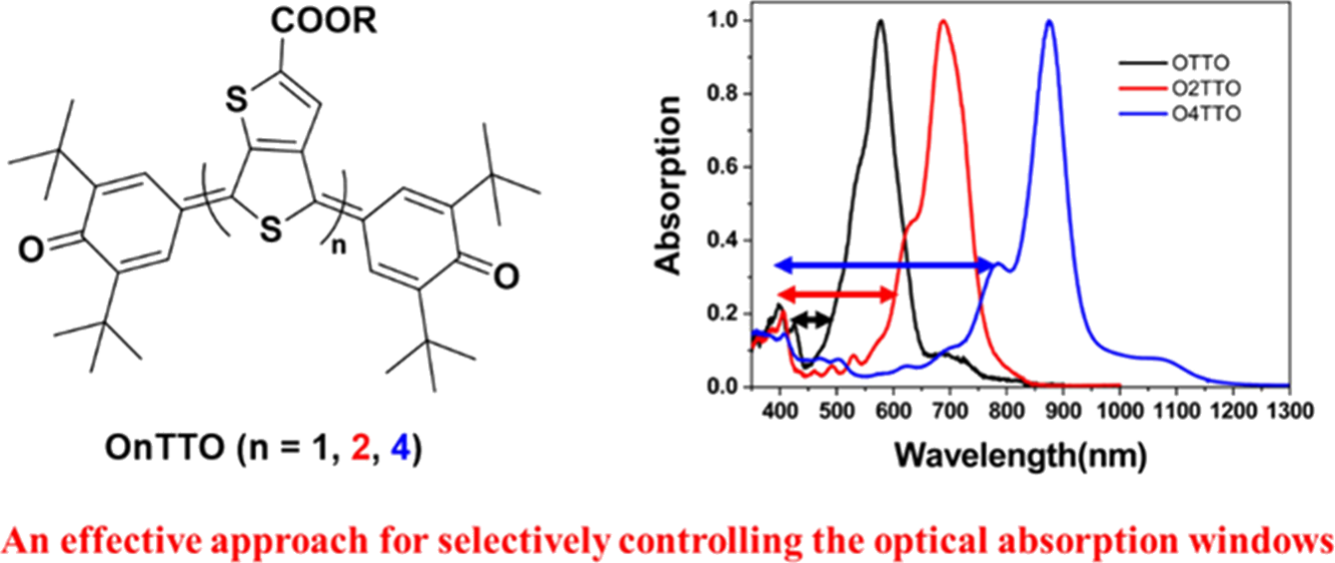

This paper describes syntheses, photophysical properties, and electrochemical characteristics of three thieno[3,4-*b*]thiophene (TT)-based quinoidal oligomers OnTTO. The rigid planar backbones of these oligomers give the molecules narrow absorption bands, and the main absorption bands were significantly red-shifted when the TT units were extended and demonstrated wide transparent windows. The compound **O4TTO** was found to possess strong absorption in the near-infrared (NIR) region approaching 1200 nm but remained transparent in the visible region. Electrochemical experiments have shown that the energy band gaps gradually narrow when the TT units are increased. Optical properties predicted by density functional theory calculations are in good agreement with the experimental optical results. These dye molecules could be promising candidates for future NIR photodetectors, filters, and bioimaging technologies.

## 1 Introduction

Organic near infrared (NIR) materials can find extensive applications in areas such as photodetectors, photofilters, heat absorbers, and bioimaging.^1,2^ Dyes with absorptions beyond 800 nm or even deeper are extensively sought after, requiring synthesis of molecules with a narrow highest occupied molecular orbital–lowest unoccupied molecular orbital (HOMO–LUMO) energy bandgap. A few classical building blocks have been intensively investigated as NIR materials for different application purposes. For instance, benzothiadiazole, isoindigo, and diketopyrrolopyrrole moieties were widely used in organic donor–acceptor (D–A) polymers and small molecules, which exhibit a narrow band gap and semiconducting properties.^3−8^ Boron-dipyrromethene and benzobisthiadiazole have been heavily studied as building blocks in biosensor and bioimaging materials due to their narrow band gap and sharp absorption peaks in the NIR region.^9−14^ It is noteworthy that our group has reported many materials exhibiting NIR absorptions, especially those containing the thieno[3,4-b]thiophene (TT) unit, which exhibit a stable quinoid structure and thus narrow bandgaps.^15,16^ Most recently, we have reported a TT-based polymer that showed a broad absorption band from 500 to 1700 nm.^17^ It is known that numerous D–A-based NIR dyes have been developed. However, selectively controlling the absorption regions remains an important and challenging task. Most of the reported D–A-based NIR materials were conjugated systems that exhibit broad absorption bands in the visible region, which would cover and obscure NIR region absorptions. These materials described here can significantly narrow the width of the absorption bands because of their planar quinoidal structures. This is important for applications like photofilters that need selective absorptions.

**Figure 1:**
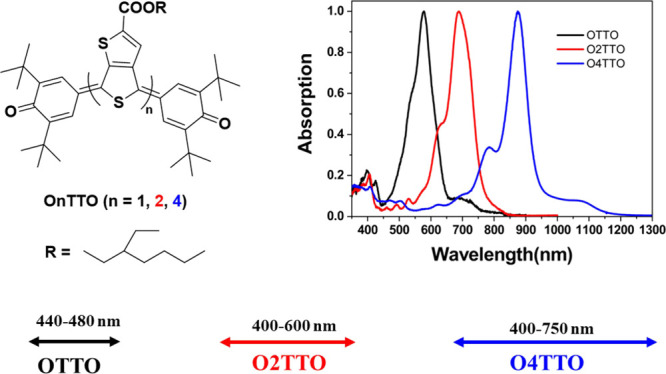
Effective approach toward selective NIR dyes.

**Scheme 1:**
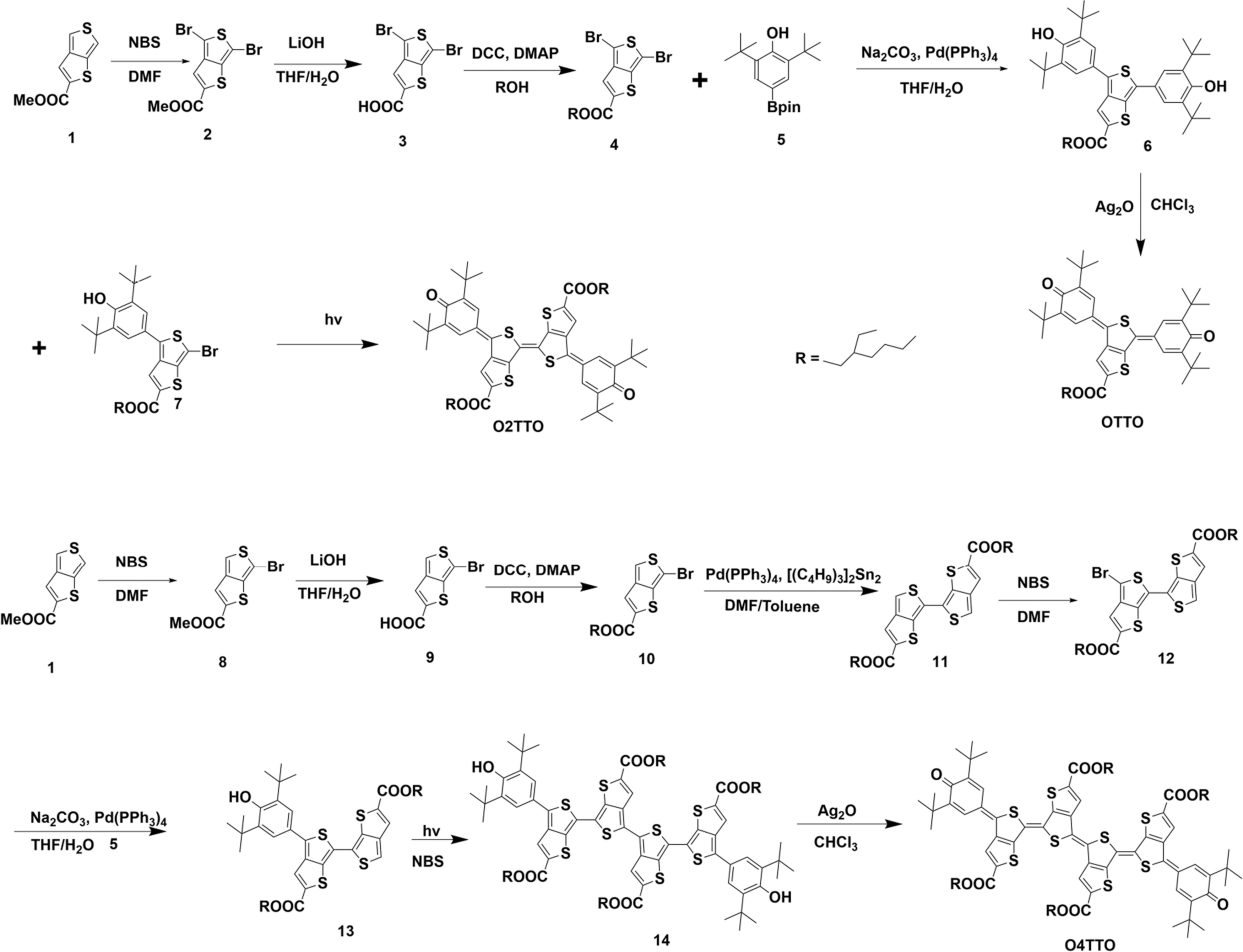
Synthetic Routes of the Quinoidal Molecules OTTO, O2TTO, and O4TTO

In this contribution, we developed a strategy to synthesize molecules that only exhibit NIR (750–1000 nm) absorption but remain transparent in the visible region (400–750 nm) (Figure 1), which is very important for applications in photodetectors and photofilters. A series of TT-based quinoidal oligomers have been rationally designed and synthesized. Since both the ester-substituted TT unit and the tert-butyl end groups can stabilize the quinoidal structure, the TT oligomers are expected to be stable quinoidal structures instead of biradicals (Scheme 1). The bandgap of the oligomers can be controlled via controlling π-delocalization.^18^ The material syntheses, structure characterization, and studies of optical, electrochemical, and electrical properties are discussed.

## 2 Results and Discussion

### 2.1 Synthesis and Characterization

The synthesis details of the quinoidal molecules are described in Scheme 1. Molecules **4** and **5** were synthesized according to reported literature procedures.^19,20^ A Suzuki coupling reaction with compound **4** and compound **5** generated two products, compounds **6** and **7**. Subsequently, the quinoidal molecule **OTTO** was oxidized from compound **6**, while the compound **O2TTO** was made directly from compound **7** through a photochemical reaction.^17^ Compound **8** was synthesized from compound **1** by monobromination, while compound **10** was synthesized via two steps that changed the alkyl group to ensure the solubility of the final quinoidal compound. The dimer thienothiophene **11** was formed by a Stille coupling reaction with compound **10**, which was monobrominated to form compound **12**. Compound **13** was produced through a Suzuki reaction between compounds **12** and **5**. Compound **14** was also isolated from the Suzuki reaction as a side product due to the self-coupling reaction of compound **13**. It can be prepared via dimerization from compound **13** with a photochemical reaction. According to the same mechanism for synthesizing **O2TTO**, bromine generated by NBS can oxidize the thienothiophene unit (compound **13**) to form a thienothiophene radical piece, followed by dimerization of the radical pieces to form the compound **14**. Finally, the target quinoidal compound **O4TTO** was oxidized from compound **13** with Ag_2_O. These compounds were fully characterized with NMR and mass spectrometry.

### 2.2 Optical Properties

**Figure 2:**
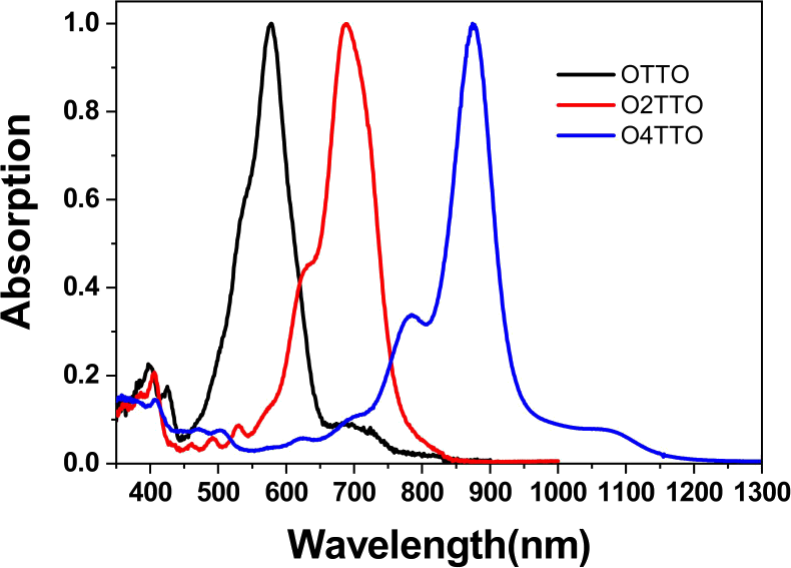
Normalized UV–vis–NIR spectra for OnTTO molecules in chloroform.

The optical properties of these three TT quinoidal molecules were investigated, the spectra are presented in Figure 2, and the detailed data are summarized in Table 1. The three compounds, **OTTO**, **O2TTO**, and **O4TTO**, dissolved in dilute chloroform solution, had very sharp absorption peaks at 578, 686, and 877 nm, respectively. Additionally, a weaker absorption peak appears at around 400 nm, which was only slightly sensitive to the modifications in the molecular structure. It is evident, however, that the main absorption peak was significantly red-shifted when the number of TT units in the molecule was increased. The absorption spectrum of **O4TTO** demonstrated a wide window from 400 to 750 nm. The **O4TTO** exhibited significant absorption in the NIR region from 750 to 1000 nm while achieving near transparency in the visible region. These results indicated that the transparent window in the visible region broadened as the number of TT units increased.

**Table 1:** Optical and Electrochemical Properties of the OnTTO Dye Molecules

dyes	in CHCl_3_ λ_max_ (nm)	solid λ_max_ (nm)	solid λ_edge_ (nm)	*E*_g_^opt^(eV)^a^	HOMO (eV)	LUMO (eV)	*E*_g_^b^ (eV)
	Abs.	Abs.	Abs.				
**OTTO**	578	571	651	1.90	–5.62	–3.97	1.65
**O2TTO**	686	652	801	1.54	–5.33	–3.98	1.35
**O4TTO**	875	803	1126	1.10	–5.00	–4.01	0.99

a Optical bandgaps were calculated based on the band edge of the solid-state absorptions.

b Redox potentials were obtained from the cyclic voltammetry (CV) measurement.

**Figure 3:**
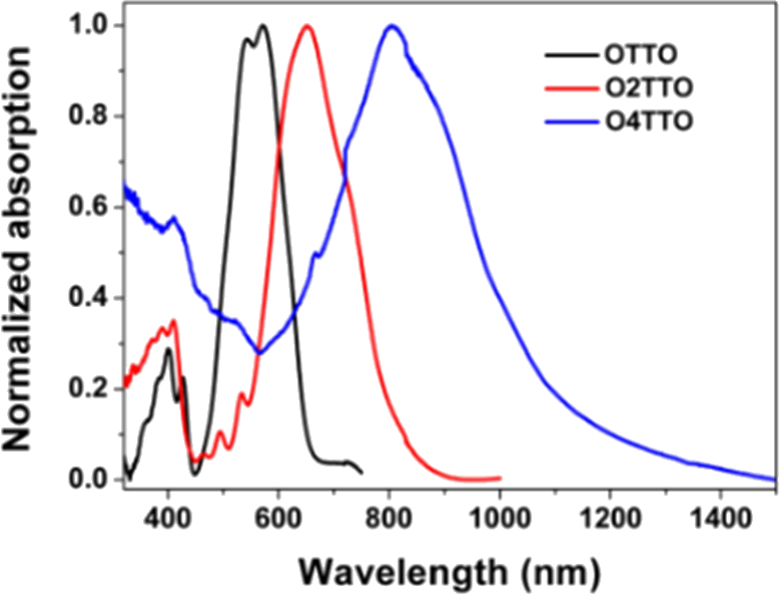
Normalized UV–vis–NIR spectra for OnTTO molecules in the solid state.

Similar spectral trends were observed in thin films. Two major peaks were observed for all three compounds (Figure 3) as in the solutions. The position of the peak around 400 nm was almost insensitive to structural modifications, while the peak in the longer wavelength region demonstrated a significant red shift as the conjugation length of the molecule increased. In the solid state, the absorption around 400 nm of the three molecules is significantly enhanced when the number of TT units is increased, which differs from the solution absorptions. The main reason is that the aggregation is relatively weak (corresponding to a lower absorption) in diluted solution; however, it is very strong in the solid state. Because of the strong aggregation in the solid state, absorption peaks show a blue shift compared to the ones in solution. The observed shift increased with the addition of TT units in the molecule, which seemed to indicate the critical role of aggregation. The optical band gaps (*E*_g_^opt^) were 1.90, 1.54, and 1.10 eV for **OTTO**, **O2TTO**, and **O4TTO**, respectively, calculated based on the band edge of the film absorptions.

Time-dependent density functional theory (TD-DFT) calculations for the molecules **O2TTO** and **O4TTO** predicted optical properties of the suggested structures, which were summarized in Figure S1. Particularly, the DFT-predicted absorption spectra had two major absorption peaks. The one around 400 nm was only slightly sensitive to the addition of extra TT units, while the one in the visible region showed a significant red shift when the conjugation length was extended. As a result, a transparent window appeared between the 400 nm peak and the main peak, a desired property.

### 2.3 Electrochemical Properties

**Figure 4:**
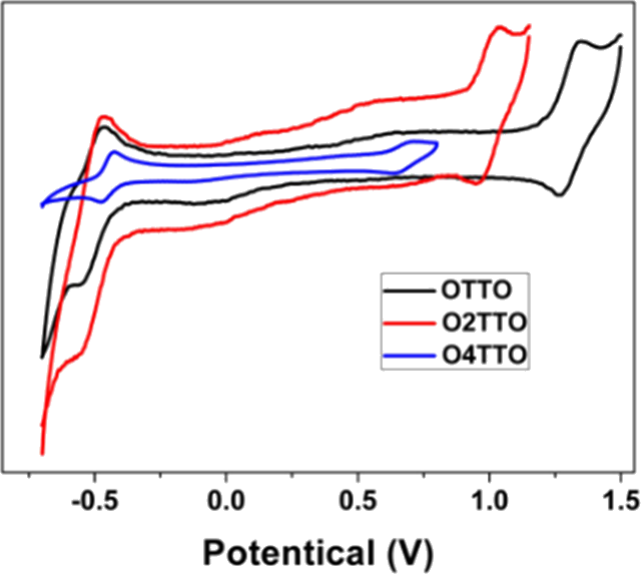
CV curves for the OnTTO molecules in CH_2_Cl_2_ solution with 0.1 M Bu_4_NPF_6_.

The electrochemical properties of the OnTTO molecules were studied by CV measurements, as shown in Figure 4, and the detailed data are summarized in Table 1. The ferrocene/ferrocenium redox couple (Fc/Fc^+^) was used as a reference which exhibited an absolute energy level of 4.8 eV to vacuum.^21^ The oxidation potential of ferrocene was 0.40 V vs SCE. The onset potentials of oxidation (*E*_ox_) for molecules **OTTO**, **O2TTO**, and **O4TTO** were observed to be 1.22, 0.93, and 0.60 V, respectively. The corresponding HOMO energy levels of **OTTO**, **O2TTO**, and **O4TTO** were −5.62, 5.33, and −5.00 eV, respectively. It was found that increasing the length of the quinoidal structure could significantly reduce the oxidation potentials due to elevation in the HOMO energy level. However, the reduction potentials of the molecules were almost the same, as the corresponding LUMO energy levels were 3.97, −3.98, and −4.01 eV for **OTTO**, **O2TTO**, and **O4TTO**, respectively. Based on these results, the energy bandgaps for **OTTO**, **O2TTO**, and **O4TTO** were 1.65, 1.35, and 0.99 eV, respectively. The electrochemical bandgaps were consistent with the optical bandgaps.

### 2.4 Electrical Properties

We further investigated electrical properties of these molecules. Charge carrier mobilities of **OTTO** and **O2TTO** were investigated with space-charge limited current (SCLC) measurements. The device fabrication details are described in the Experimental Section. Mobility was derived from fitting the SCLC region of our I-V curve as per the Mott–Gurney equation  where *J* – current density, μ – mobility, ε – dielectric constant, *V* – effective voltage, and *L* – thickness of the film. These results are summarized in Table 2.

**Table 2:** Hole and Electron SCLC Mobility of **OTTO** and **O2TTO**

system	μ_hole,_ (cm^2^V^–1^ s^–1^) × 10^–5^	μ_el,_ (cm^2^V^–1^ s^–1^) × 10^–5^
**OTTO**	0.6 ± 0.1	4.6 ± 1.4
**O2TTO**		5.6 ± 1.3

For both **OTTO** and **O2TTO** molecules, the observed SCLC mobility was found to be around 10^–5^–10^–6^ cm^2^V^–1^ s^–1^. This is a rather small value, comparable to the mobilities of other electron-deficient semiconducting materials, such as those based on PDI synthesized by our group in previous research. These materials showed promise in OPV devices.^22,23^ For **O2TTO**, however, we could not measure the electrical properties of hole-only devices due to excessive aggregation of **O2TTO** on PEDOT: PSS upon drying, which led to shortening of the device. The same situation also happened for **O4TTO**.

## 3 Conclusions

A series of TT-based quinoidal oligomers (**OTTO**, **O2TTO**, and **O4TTO**) were successfully synthesized and systematically characterized. By carefully investigating the optical properties of the three molecules, we demonstrated an effective method for finely tuning the energy levels and selectively and precisely controlling the optical absorption windows of the molecules. Molecule **O4TTO** showed interesting absorption bands in solution, including a sharp absorption peak in the NIR region from 750 to 1000 nm and near transparency in the visible region from 400 to 750 nm. Thus, it showed potential in certain applications, such as NIR photodetectors and NIR filters. The present study paved the way for the development of novel NIR dyes with designated absorptions for specified applications. Further molecular structure modifications are underway.

## 4 Experimental Section

NMR spectra were collected on a Bruker DRX-500 spectrometer. MALDI-TOF and GC–MS were used for molecular mass measurements. The C, H, and S elemental contents of the quinoidal compounds were determined by elemental analysis. A SHIMADZU UV-3600 spectrometer was used for measuring UV–vis–NIR absorptions. CV measurements were carried out by using Pt as working and counter electrodes, and Ag/AgCl was used as the reference electrode, in a CH_2_Cl_2_ solution with 0.1 M Bu_4_NF_6_. As for the charge carrier mobility, vertically stacked device: ITO/ZnO/active layer/Ca/Al and device: ITO/PEDOT: PSS/active layer/MoO_3_/Ag were used for electron-only and hole-only measurements, respectively.

All chemicals were bought and used directly. Toluene and THF were dried with sodium prior to use for water-sensitive reactions. Detailed syntheses and structural characterization of the TT-based oligomers are in the Supporting Information.

## Supporting Information Available

DFT calculations for optical properties and synthesis of the dye molecules (PDF)
