# Supplementary material for: Effective Approach toward Selective Near-Infrared Dyes: Rational Design, Synthesis, and Characterization of Thieno[3,4-b]thiophene-Based Quinoidal Oligomers
Source: ACS Appl Mater Interfaces. 2022 Dec 12;14(50):55686–90. doi: 10.1021/acsami.2c18633 (PMC9782366; doi:10.1021/acsami.2c18633)
Supplement: Supplementary file 1 — am2c18633_si_001.pdf [file am2c18633_si_001.pdf]

Supporting Information

An Effective Approach towards Selective Near Infrared Dyes: Rational Design, Synthesis, and Characterizations of Thieno[3,4-b]thiophene Based Quinoidal Oligomers

Yuxuan Hei,[a] Xinwei Zhang,[a] Pengxing He,[a] Eric Jiahan Zhao,[b] Edison Tang,[b] Valerii Sharapov,[b] Xunshan Liu,[a,b,]* Luping Yu[b,]*

[a] *Key Laboratory of Surface & Interface Science of Polymer Materials of Zhejiang Province，Department of Chemistry, Zhejiang Sci-Tech University, 928 Second Street, Hangzhou, 310018, China*

[b] *Department of Chemistry and the James Franck Institute, The University of Chicago, 929 E57th Street, Chicago, Illinois, 60637, United States*

* Corresponding author: xliu350@zstu.edu.cn; lupingyu@uchicago.edu

Theoretical predication

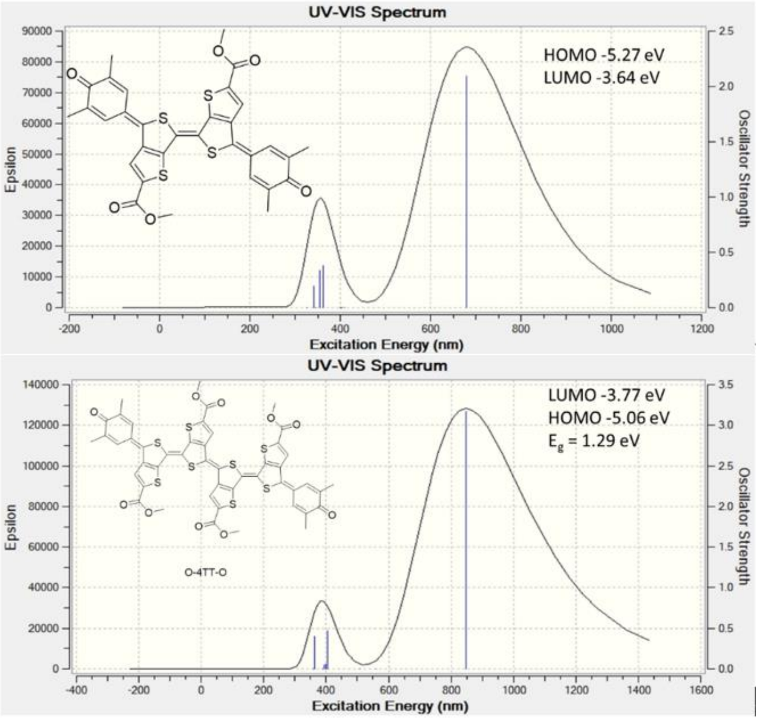

Figure S1 UV-Vis absorption spectra of the **O2TTO** and **O4TTO** in chloroform solution predicted from the TD-DFT calculations

There are many reported quinoidal molecules are end-capped with -C(CN)$_2$ groups, we have actually also thought about this, and made DFT calculations for the structures as shown below (**Figure S2**), which indicated that the energy gap will be further reduced and the main absorption pick will be more red-shifted compare to the molecule O4TTO, whereas because of the strong electron withdraw ability of the -C(CN)$_2$ group, intramolecular charge transfer caused absorptions will definitely appeared in the visible region. Thus, to keep the visible region transparent, we did not prepare the molecules with -C(CN)2 end groups in this work.

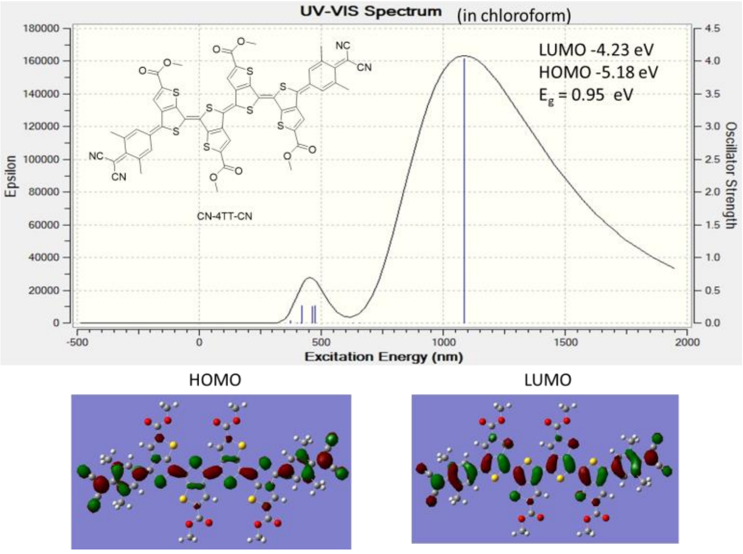

Figure S2 UV-Vis absorption spectra of the reference molecule end with -C(CN)$_2$ group in chloroform solution predicted from the TD-DFT calculations

Syntheses of the compounds and ^{1}H NMR spectra of the dye molecules OnTTO

2-ethylhexyl-6-bromo-4-(3,5-di-tert-butyl-4-hydroxyphenyl)thieno[3,4-b]thiophene-2-carboxylate (6)

2-ethylhexyl-4,6-bis(3,5-di-tert-butyl-4-hydroxyphenyl)thieno[3,4-b]thiophene-2-carboxylate (7)

Under an inert atmosphere, a mixture of compound **4** (908 mg, 2.0 mmol), **5** (664 mg, 2.0 mmol), $Pd(PPh_3)_4$ (115 mg, 0.1 mmol), and sodium carbonate (1.06 g, 10 mmol) in 30 mL of THF and 5 mL of H_2O was stirred at 85 °C for 24 h. The reaction was cooled down to room temperature and the reaction mixture was extracted with dichloromethane (DCM) 3 times. The combined organic phase was washed with water (2 x 20 mL) and dried over $MgSO_4$. The solvent was evaporated under vacuum and the residue was purified by silica gel chromatography (DCM/Hexane 1/3), affording crude compound **6** (135 mg, 12%) and pure compound **7** (250 mg, 18%), with a total yield of 30%.

Compound **6** was not stable under room light. Characterizations for the pure compound was not recorded.

Compound **7** (250 mg 18%). ^{1}H NMR (500 MHz, $CDCl_3$ δ/ppm): 7.88 (s, 1H), 7.53 (s, 2H), 7.49 (s, 2H), 5.39 (s, 1H), 5.34 (s, 1H), 1.53 (s, 18H), 1.52 (s, 18H); MS (Maldi-tof): *m/z* calcd for $C_{43}H_{60}O_4S_2$: 705.1; found: 704.5.

Methyl 6-bromothieno[3,4-b]thiophene-2-carboxylate (8) Compound **1** (3.96 g, 20.0 mmol) was dissolved in 100 mL of anhydrous N,N-Dimethylformamide (DMF). The *N*-bromosuccinimide (NBS) (3.56 g, 20 mmol) dissolved in 80 mL DMF was added to the solution dropwise at 0 °C over 3h. The reaction mixture was stirred at 0 °C for 2 h in darkness. Then the mixture was poured into water and extracted with DCM. The organic phase was dried over anhydrous $MgSO_4$, and the solvent was subsequently evaporated under vacuum. The crude product was purified on silica gel chromatography using a hexanes/DCM mixture (1/1 by volume) as eluent to afford a pink solid (4.32 g, 78%). The solid contains two mono-brominated isomers. According to ^{1}H NMR, the ratio of the two monomers was close to 1:1. The monomers could not be further isolated with silica gel chromatography. ^{1}H NMR (500 MHz, $CDCl_3$, δ/ppm): 7.67 (s, 1H), 7.58 (d, 1H), 7.53 (s, 1H), 7.25 (d, 1H), 3.92 (s, 6H). GC-MS ($C_8H_5BrO_2S_2$) m/z: calcd for 277.2; found 277.8.

6-bromothieno[3,4-b]thiophene-2-carboxylic acid (9) To a solution of compound **8** (4.00 g. 14.4 mmol) in THF/H_2O (360 mL, 2/1) was added LiOH•H_2O (2.92 g. 69.5 mmol) at room temperature. The reaction mixture was refluxed for 6 h. After cooling to room temperature, THF was evaporated under vacuum. The residue was acidified with 1M HCl and filtered to afford pale yellow solids (3.65 g, 96%). The isomer mixture was directly used for the next step without further purification. ^{1}H NMR (500 MHz, $CDCl_3$, δ/ppm): 8.03 (s, 1H), 7.81 (s, 2H), 7.76 (s, 1H), 7.46 (s, 3H). GC-MS ($C_7H_3BrO_2S_2$) m/z: calcd for 263.12; found: 263.8.

2-ethylhexyl 6-bromothieno[3,4-b]thiophene-2-carboxylate (10) To a solution of compound **9** (263.1 mg. 1.0 mmol), DCC (247.4 mg, 1.2 mmol) and DMAP (146.6mg, 1.2 mmol) in DCM (5 mL) was added 2-ethylhexan-1-ol (0.79 mL, 5 mmol) at room temperature. The reaction mixture was stirred for 24 h under N_2 protection.

Subsequently, the reaction was diluted with DCM (30 mL), washed with water (10 mL) and brine (10 mL), and dried over anhydrous Na_2SO_4. The organic solvents were evaporated under vacuum and the residue was purified by column chromatography (silica gel, DCM/hexane = 2:3) to afford compound **10** (178 mg 47%) as a colorless oil.

Compound **10** ^{1}H NMR (500 MHz, $CDCl_3$ δ/ppm): 7.55 (d, 1H), 7.24 (d, 1H), 4.24 (m, 2H), 1.71 (m, 1H), 1.47-1.30 (m, 8H), 0.96-0.88 (m, 6H). ^{13}C NMR ($CDCl_3$ δ/ppm): 163.0, 146.1, 141.0, 139.1, 122.5, 112.8, 103.0, 68.4, 39.0, 30.7, 29.2, 24.1, 23.2, 14.3, 11.3. GC-MS ($C_{15}H_{19}BrO_2S_2$) m/z: calcd for 375.3; found: 375.9.

Bis(2-ethylhexyl) [6,6'-bithieno[3,4-b]thiophene]-2,2'-dicarboxylate (11) Under an inert atmosphere, a mixture of compound **10** (1.2 g, 3.2 mmol), Bis(tributyltin) (2.5 g, 4.3 mmol), $Pd(PPh_3)_4$ (180 mg, 0.16 mmol) in 10 mL of anhydrous toluene and 10 mL of DMF was stirred at 100 °C for 24 h. The reaction was quenched with 10 mL H_2O. The mixture was then extracted with DCM 3 times. The combined organic phase was washed with water (2 x 20 mL) and dried over $MgSO_4$. The solvent was evaporated and the residue was purified by silica gel (DCM/Hexane 3/2) affording the title compound as a red solid (0.84 g, 89%). ^{1}H NMR (500 MHz, $CDCl_3$ δ/ppm): 8.00 (d, 2H), 7.30 (d, 2H), 4.26 (m, 4H), 1.73 (m, 2H), 1.47-1.31 (m, 16H), 0.97-0.90 (m, 12H); ^{13}C NMR ($CDCl_3$ δ/ppm): 162.8, 142.0, 140.8, 140.7, 127.2, 123.5, 110.9, 68.2, 38.8, 30.5, 29.0, 24.0, 23.0, 14.1, 11.1; MS (Maldi-tof): *m/z* calcd for $C_{30}H_{38}O_4S_4$: 590.9; found: 590.2.

Bis(2-ethylhexyl)4-bromo-[6,6'-bithieno[3,4-b]thiophene]-2,2'-dicarboxylate (12) Compound **11** (472 mg, 0.8 mmol) was dissolved in 25 mL of DMF, NBS (142 mg, 0.8 mmol) dissolved in 15 mL DMF was added to the solution dropwise at 0 ^{o}C over 1h. The reaction mixture was stirred at 0 ^{o}C for 2 h in the dark. Then the mixture was poured into water and extracted with dichloromethane. The organic phase was dried over anhydrous $MgSO_4$, and then the solvent was evaporated under vacuum. The crude product was purified on silica gel chromatography using a DCM/Hexanes mixture (3/2 by volume) as eluent to isolate a red solid **12** (365 mg, 68%). ^{1}H NMR (500 MHz, $CDCl_3$ δ/ppm): 7.77 (s, 1H), 7.71 (s, 1H), 7.14 (s, 1H), 4.22 (m, 4H), 1.71 (m, 2H), 1.45-1.33 (m, 16H), 0.97-0.92 (m, 12H); ^{13}C NMR ($CDCl_3$ δ/ppm): 162.7, 162.4, 142.3, 141.8, 141.2, 141.0, 140.9, 140.5, 128.7, 126.2, 124.2, 123.0, 111.2, 97.4, 68.4, 68.4, 38.9, 38.9, 30.7, 30.6, 29.1, 29.1, 24.1, 24.0, 23.1, 23.1, 14.3, 14.2, 11.2, 11.2; MS (Maldi-tof): *m/z* calcd for $C_{30}H_{37}BrO_4S_4$: 669.8; found: 669.7.

Bis(2-ethylhexyl)-4-(3,5-di-tert-butyl-4-hydroxyphenyl)-[6,6'-bithieno[3,4-b]thiophene]-2,2'-dicarboxylate (13) Under an inert atmosphere, a mixture of compound **12** (200 mg, 0.3 mol), **5** (200 mg, 0.6 mmol), $Pd(PPh_3)_4$ (70 mg, 0.06 mmol), and sodium carbonate (159 g, 1.5 mmol) in 15 mL of THF and 1.5 mL of H_2O was stirred at 85 °C for 24 h. The reaction was cooled down to room temperature and the reaction mixture was extracted with DCM 3 times. The combined organic fractions were washed with water (3 x 20 mL) and dried over $MgSO_4$. The solvent was evaporated under vacuum and the residue was purified by silica gel chromatography (DCM/Hexane 1/1), affording compound **13** (203 mg, 85%) as a red solid. ^{1}H NMR (500 MHz, $CDCl_3$ δ/ppm): 8.09 (d, 1H), 8.03 (s, 1H), 7.57 (s, 2H), 7.23 (d, 1H), 5.45 (s, 1H), 4.31-4.25 (m, 4H), 1.79-1.72 (m, 2H), 1.56 (s, 18 H), 1.38-1.26 (m, 16H), 0.94-

0.88 (m, 12H); ^{13}C NMR ($CDCl_3$ δ/ppm): 162.78, 162.7, 153.8, 142.5, 140.2, 140.0, 134.5, 130.5, 127.6, 124.4, 123.7, 122.9, 110.1, 83.1, 68.1, 67.9, 38.7, 38.7, 34.4, 31.4, 30.4, 30.3, 28.8, 28.8, 23.9, 23.9, 22.8, 22.8, 13.9, 13.9, 11.0, 11.0; MS (Maldi-tof): *m/z* calcd for $C_{40}H_{58}O_5S_4$: 795.2; found: 794.1.

2-ethylhexyl-4,6-bis(3,5-di-tert-butyl-4-oxocyclohexa-2,5-dien-1-ylidene)-4,6-dihydrothieno[3, 4-b]thiophene-2-carboxylate (OTTO)

Compound **7** (70 mg, 0.1 mmol) was dissolved in 5 mL $CHCl_3$, Ag_2O (232 mg, 1 mmol) was added to the solution. The solution was then stirred at room temperature over 12 h, followed by a filtration to remove the unreacted Ag_2O. The residue was purified by silica gel (DCM/Hexane 1/1), affording compound **OTTO** (43 mg, 61%) as a purple solid. ^1H NMR (500 MHz, $CDCl_3$ δ/ppm): 8.20 (s, 1H), 7.79 (s, 1H), 7.67 (s, 1H), 7.30 (s, 2H), 4.38-4.29 (m, 2H), 1.77-1.71 (m, 1H), 1.50-1.26 (m, 44H), 0.98-0.88 (m, 6H) (Fig.1); MS (Maldi-tof): *m/z* calcd for $C_{43}H_{58}O_4S_2$: 703.1; found: 703.4. Elem. Anal. Calcd. for ($C_{43}H_{58}O_4S_2$) (%): C, 73.46; H,8.32; S, 9.12; Found (%): C, 73.29; H, 8.41; S, 9.04.

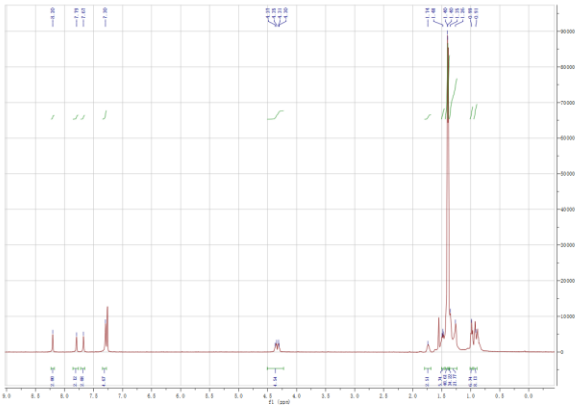

Figure S3 ^1H NMR spectrum for compound OTTO

Bis(2-ethylhexyl)(E)-4,4'-bis(3,5-di-tert-butyl-4-oxocyclohexa-2,5-dien-1-ylidene)-4H,4'H-[6,6'-bithieno[3,4-b]thiophenylidene]-2,2'-dicarboxylate (O2TTO) Compound was obtained directly by exposing compound **6** to room light, without the addition of any other external reagents. The product was purified by silica gel chromatography (DCM/Hexane 2/1), affording compound **O2TTO** as a blue solid. ^1H NMR (500 MHz, $CDCl_3$ δ/ppm): 8.18 (s, 2H), 7.64 (s, 2H), 7.28 (s, 2H), 4.39-4.32 (m, 4H), 1.80-1.75 (m, 2H), 1.51-1.37 (m, 52H), 1.02-1.00 (t, 6H), 0.94-0.92 (t, 6H) (Fig.2); MS (Maldi-tof): *m/z* calcd for $C_{58}H_{76}O_6S_4$:997.5; found: 997.5. Elem. Anal. Calcd. for ($C_{58}H_{76}O_6S_4$) (%): C, 69.84; H,7.68; S, 12.86; Found (%): C, 69.65; H, 7.77; S, 12.76.

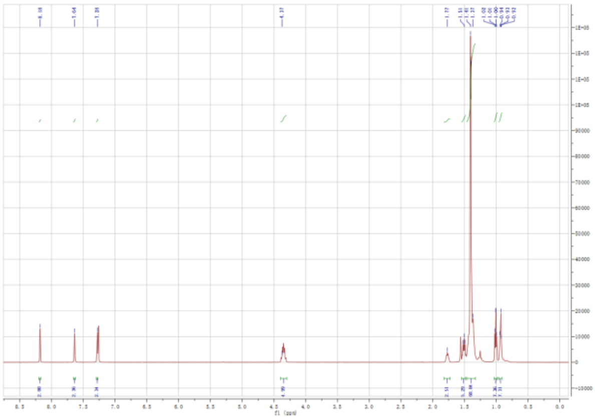

Figure S4 ^{1}H NMR spectrum for compound O2TTO

Tetrakis(2-ethylhexyl)(4'E,6E,6''E)-4,4'''-bis(3,5-di-tert-butyl-4-oxocyclohexa-2,5-dien-1-ylidene)-4H,4'''H-[6,6':4',4'':6'',6'''-quaterthieno[3,4-b]thiophene]-2,2',2'',2'''-tetracarboxylate (O4TTO) Compound **13** (50 mg, 0.06mmol) was dissolved in 5 mL hexanes, NBS (33 mg, 0.19 mmol) was added to the solution. The solution was sealed in a glass vial with N_2 protection. Then the reaction was stirred under a 500 W Mercury lamp for 3 Min. The solvents of the obtained green solution were evaporated under vacuum, and the crude product was further purified by silica gel chromatography using DCM as eluents. The target product **O4TTO** was obtained as a green solid (35mg, 70%). ^{1}H NMR (500 MHz, $CDCl_3$ δ/ppm): 8.01 (s, 2H), 7.93 (s, 2H), 7.51 (s, 2H), 7.17 (s, 2H), 4.45-4.6 (m, 8H), 1.92-0.88 (m, 96H) (Fig. 3); MS (Maldi-tof): *m/z* calcd for $C_{88}H_{112}O_{10}S_8$: 1586.3; found: 1586.2. Elem. Anal. Calcd. for ($C_{88}H_{112}O_{10}S_8$) (%): C, 66.63; H,7.12; S, 16.17; Found (%): C, 66.47; H, 7.21; S, 16.08.

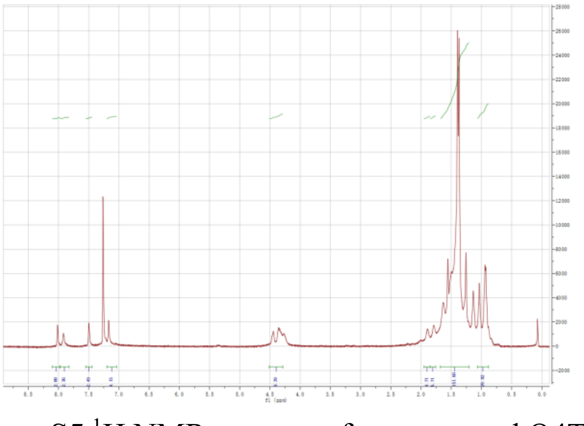

Figure S5 ^{1}H NMR spectrum for compound O4TTO
